# Using interactive SMS support groups to prevent mother-to-child HIV transmission in South Africa: a qualitative analysis of social and psychological benefits

**DOI:** 10.1080/17290376.2021.1989323

**Published:** 2025-08-22

**Authors:** Cindy A. Crusto, Samantha Pittenger, Jessica Costeines, Ndidiamaka Amutah-Onukagha, Anna Kydd, Maretha Visser, Thu Do, Andrea Dean, Brian Forsyth

**Affiliations:** a Keck School of Medicine of the University of Southern California, Los Angeles, USA; b Department of Psychiatry, Yale University School of Medicine, New Haven, CT, USA; c Department of Psychology, University of Pretoria, Pretoria, South Africa; d Department of Public Health and Community Medicine, Tufts University School of Medicine, Boston, MA, USA; e SHM Foundation, London, UK; f Ember Mental Health, London, UK; g Gilead Sciences, Boston, USA; h Department of Pediatrics, Baylor College of Medicine/Texas Children’s Hospital, Houston, TX, USA; i Department of Pediatrics, Yale University School of Medicine, New Haven, CT, USA; j Center for Interdisciplinary Research on AIDS, Yale School of Public Health, New Haven, CT, USA

**Keywords:** PMTCT, SMS support group intervention, mobile health, social support

## Abstract

Although advances have been made in the prevention of mother-to-child transmission (PMTCT) of HIV, social and psychological factors associated with learning of an HIV diagnosis and living with HIV during pregnancy can impact medication adherence and health outcomes. Mobile technology can increase social support; however, it is unclear if such technology can be used to provide social support to reduce negative psychological outcomes. This study analysed the feasibility of conducting a multi-way interactive SMS-based support group with HIV-positive women in the PMTCT programme in clinics of Tshwane, South Africa. We studied the types of social support women received through using this mobile technology to address personal, interpersonal and social barriers to PMTCT adherence. We analysed the social and psychological content of SMS messages sent between seven HIV-positive pregnant women participating in a 12-week interactive support group. We conducted a qualitative, thematic analysis of the 734 text messages. Five social/psychological themes emerged: appraisal and emotional support, informational support, spiritual support, acceptance and disclosure of HIV status, and gratefulness for the support group, suggesting benefits of the interactive SMS-based support group. This interactive support model can provide social support and information to pregnant women living with HIV, leading to a reduction in isolation and negative psychological outcomes which could promote positive health outcomes.

Approximately 30.7% of pregnant women aged 15–49 years in South Africa are living with HIV (Woldesenbet et al., 2019). Important advances have been made in the prevention of mother-to-child transmission (PMTCT) of HIV as a means of decreasing the HIV incidence among children of HIV-positive women (SA National Department of Health, 2019). PMTCT programmes are widely available, yet there are high levels of non-adherence and/or disengagement from care during pregnancy (Phillips et al., 2014; Weiss et al., 2014). Failure of the PMTCT programme occurs at several levels (Rispel, Peltzer, Phaswana-Mafuya, Metcalf, & Treger, 2009) including personal (such as low self-efficacy and negative affect), interpersonal (e.g. lack of male involvement, limited social support), social (e.g. stigma, fear of disclosure) and service delivery barriers (Colvin et al., 2014; Hodgson et al., 2014; Omonaiye, Kusljic, Nicholson, & Manias, 2018). Especially women diagnosed during pregnancy were vulnerable and needed support to adhere to treatment (Omonaiye et al., 2018; Phillips et al., 2014).

The World Health Organization (WHO, 2008) recommends peer support groups to increase retention and adherence in treatment for HIV, decrease feelings of isolation and stigma, and promote individuals’ self-esteem (WHO, 2008). Support group interventions for South Africans living with HIV have resulted in decreased internal HIV-related stigma, higher rates of status disclosure, increased treatment adherence, reduced mortality and morbidity (Bateganya, Amanyeiwe, Roxo, & Dong, 2015; Mundell et al., 2011; Tumwikirize & Mokoboto-Zwane, 2016).

The past decade has seen an increase in the use of mobile technology to promote contact with health consumers and promote medical regimen adherence for HIV-positive individuals (Catalani, Philbrick, Fraser, Mechael, & Israelski, 2013; Hirsch-Moverman et al., 2017). These programmes capitalise on the automation, convenience, and anonymity of mobile phone messaging and applications to provide appointment reminders, collect information and disseminate information about HIV and treatment. Mobile phone technology can also be used to increase social support and interaction among people living with HIV, while reducing the experience of stigma (Blaya, Fraser, & Holt, 2010). There has not been research to date on how effective mobile technology is in providing interactive support for pregnant women with HIV to address the personal, social, and interpersonal barriers to treatment adherence.

In 2012, a group of researchers conducted a pilot study to assess the feasibility of a multi-way interactive peer social support programme administered via text message with the goal of increasing PMTCT programme adherence among HIV-positive women in rural South Africa (Dean, Makin, Kydd, Biriotti, & Forsyth, 2012). Dean et al. (2012) reported on the medical knowledge participants gained from their participation in the SMS support group. The purpose of the present study was to examine the personal, social, and interpersonal themes that arose during this 12-week multi-way interactive SMS support group.

In the analysis the social support model of Simoni, Frick, and Huang (2006) was used as a framework. The model identified four types of social support important to promoting ART adherence: Appraisal, Emotional, Informational, and Spiritual support. Appraisal support involves actions by others that serve to increase one’s self-efficacy, such as encouragement, affirmation, and modelling of adaptive behaviours. Emotional support serves to reduce negative affect through empathy and expressions of care from one’s social network. Pregnant women living with HIV experience elevated rates of depressive symptoms and suicidal ideation (Rodriguez, Cook, Peltzer, & Jones, 2017), making emotional support particularly important to this population. Indeed, pregnant women living with HIV are more likely to endorse suicidal ideation and behaviour when they perceive a lack of social support (Onah, Field, Bantjes, & Honikman, 2017) and when they experience AIDS-related stigma (Rodriguez et al., 2017). Conversely, HIV-positive individuals who participate in support groups report fewer depressive symptoms compared to those who do not participate (Bateganya et al., 2015). Informational support enhances knowledge of HIV and ART through provision of facts. Finally, spiritual support (e.g. joining in prayer; acknowledgement of greater purpose) may also strengthen coping and adjustment to HIV diagnosis (Simoni et al., 2006). Supporting this theory, Simoni et al. (2006) found that perceived social support reduced negative affect, enhanced spirituality, and built self-efficacy thus increasing ART adherence.

To shed light onto the potential influence of SMS communications on decreasing the barriers to treatment adherence, we analysed the social and psychological content of text messages between pregnant women participating in a mobile interactive support group.

## Methods

### Research design

This study used a qualitative design to explore if the mobile interactive peer support intervention can provide HIV-infected pregnant women with the support they need to address personal, social and interpersonal barriers to PMTCT treatment adherence. We analysed the content of the text messages women sent as part of the support group interaction.

### Participants

A purposive sample was selected through recruiting participants from two prenatal clinics located in urban townships of Tshwane (Pretoria) in South Africa. The participants were selected because of their relevant experience to the research problem (Creswell, 2014). Women were eligible to participate in the SMS support group if they (1) were 18 years of age or older, (2) were pregnant (estimated to be between 15 and 33 weeks’ gestation as determined by clinic staff at the start of the intervention), (3) were comfortable writing and speaking in English, (4) were diagnosed HIV-positive for the first time during the current pregnancy, and (5) had home access to electricity to charge a mobile phone. Healthcare workers identified eligible participants and referred them to the researcher who explained the intervention in detail.

Twelve women were referred as eligible to participate in the study; however, the researchers regarded one woman as ineligible due to her inability to communicate in English. Four women wanted more time to consider participating but did not return to the clinic during the enrolment period. Thus, seven women participated in the study. Although the sample was small, based on our prior experience with support groups, six to 10 participants was considered an appropriate size for the pilot group to assess feasibility of the intervention (Mundell, Visser, Makin, Forsyth, & Sikkema, 2012).

The mean age of the participants was 26 years. Participants spoke five primary languages and the group was conducted in English. All participants’ level of education ranged from completion of seventh grade to high school. One participant was employed full time, while the others were seeking employment. All participants reported having a romantic partner and four reported cohabitating with their partner. At the start of the intervention, five out of the seven participants reported having disclosed their positive HIV status to at least one person (two to sister and partner, two to partner only, and one to a family member). Only one participant was certain that her romantic partner had been tested and was HIV-positive, three were unsure if their partner had been tested, and three reported that their partners had not been tested. Three participants were taking ART at the start of the support group. Of the remaining four participants, three were waiting to begin medication and one declined medication. One participant discussed thoughts of self-harm in the SMS interaction, and the researcher consulted with a physician on the research team who advised a referral. The participant’s clinical team was immediately contacted, and they reached out to her to assess her wellbeing. Additionally, she was seen in her clinic immediately, removed from the study, and referred to psychiatric care.

### Intervention: Project Kopano

Project Kopano was a pilot project designed, developed, and run by the SHM Foundation – the charitable arm of London-based consulting house SHM Productions – in partnership with the Yale School of Medicine and the University of Pretoria to provide an SMS-based support group to pregnant women living with HIV in Pretoria, South Africa. Project Kopano was informed by Project Zumbido, a pilot project in Mexico that provided 40 adult participants from rural and urban areas of the state of Jalisco with mobile phones and unlimited text messages (Mapham, 2008).

Project Kopano was developed using the Information – Motivation – Behavioural Skills (IMB) model of HIV/AIDS preventive behaviour (Fisher & Fisher, 2000). The IMB model posits that having information regarding the transmission and prevention of HIV/AIDS is necessary for preventive behaviours to follow. Secondly, individuals must be motivated to engage in behaviours that prevent the transmission of HIV/AIDS. Last, individuals must have the skills necessary to engage in preventive behaviours. Following this model, the interactive SMS group could provide information regarding what pregnant HIV-positive women should do to remain healthy. Additionally, through addressing attitudes toward performing specific HIV preventive acts, perceptions of social support to engage in HIV prevention behaviours, and behavioural intentions to engage in HIV-preventive behaviours (Ajzen & Fishbein, 1980), the SMS group could provide the motivation for behavioural change. And finally, on a social level, the SMS group provides group acceptance, which leads to motivation of group behaviour to adhere to PMTCT.

Project Kopano provided HIV-infected women who were part of the project with basic and inexpensive mobile phones to connect with other participants. Some women did not have phones of their own or shared cell phones with their partners/families. Participants were asked to send text messages pertaining to any medical or psychosocial questions, concerns, or ideas via the study cell phone to interact with each other and provide both advice and support. Participants were encouraged to respond to their peer’s text messages or simply observe the back and forth conversations according to their level of comfort with the given topic. Unlike typical in-person support groups, communication between the participants was generally unstructured and free-flowing conversations solely through text messages. These conversations could occur at any time of the day or night. Although participants had the ability to turn their phones off at any given time, they were encouraged to keep it on and silenced when needed. This allowed participants to receive all SMS messages and review them when convenient. Participants remained anonymous from each other throughout the intervention. The participation agreement did not include what could or could not be discussed because the agreement addressed the use of the cell phone for group purposes only, to protect the participants’ identity and health status. It was an ethics consideration to keep the study anonymous and to make women feel they could be open and not trackable in any way.

The SMS-based support group intervention, study methods and technology used for Project Kopano, have been described previously (see Dean et al., 2012).

The text message support group was comprised of two facilitators: a clinician/researcher and a peer mentor. The clinician had ready access to physicians who were available to answer any questions related to HIV/AIDS, obstetrics and gynaecological issues, and general physical health. They also monitored SMS activity to correct inaccurate information and to guide conversation using a checklist of relevant topics which were adapted from a structured support group developed for HIV-positive South African women (Visser, Mundell, De Villiers, Sikkema, & Jeffery, 2005). The facilitators were available 24 h a day, and texted within 30 min to correct any misinformation shared among the participants. If there was a lull in text messages (i.e. more than one day with an unanswered question, more than one week without a text, or more than three texts in row from one person without any response) the clinician was responsible for restarting the conversation amongst participants.

The peer mentor – who was a mother who had participated in PMTCT herself – was available on the group messaging system to provide further support as necessary. While the participants talked with and educated each other by sharing their own knowledge and experiences, the facilitators acted as bystanders but were available to answer any questions that arose while guiding and encouraging group discussion. The facilitators also were responsible for tracking and contacting a participant if the participant used the mobile phone for reasons not related to the project or if they violated their Participation Agreement, which ensured group confidentiality, respect towards one other, and contributions to the group at a level with which they were comfortable.

*Technology*: Each participant was provided with a very basic and inexpensive mobile phone that was credited with prepaid airtime and unlimited SMS capabilities. A brief instructional session as well as a handout was disseminated to familiarise participants on how to use the study phone, although most participants were familiar with mobile technology. Strict instructions were given by the researchers to use the phone only for the project. To protect the privacy of participants, the researcher advised personal and private access to the phones and how to explain to their families that they now had a phone. The women decided they would tell their family they had been invited to be part of a support group about pregnant women. At any time, participants could stop participating in the study because the phones and SIM cards belonged to the study, and participants could simply stop using the phone or replace the SIM card.

Because of the sensitivity of the topic several safeguards were enacted to protect participants’ privacy, including registering mobile phones and SIM cards to the researcher (eighth author, A.D.). Participants were asked to create and use a ‘nickname’ that allowed participants to remain anonymous, and phone numbers were unable to be extracted. Each participant was encouraged to use their PIN number to assure privacy of messages. Participants were told that if they were concerned that their messages might be read by someone else, that they should think about deleting their messages regularly from their inbox and sent box. They were asked to use a username when interacting in the group to assure privacy and anonymity. The participant agreement emphasised the importance to respect confidentiality in the group. In their first SMS session they discussed issues of confidentiality.

The group SMS format was made possible with ZygoHubs© and BulkSMS© technology. ZygoHubs© was a United Kingdom-based group text messaging platform that allows several individuals to communicate via SMS using a single mobile phone number. For the purposes of this project, ZygoHubs© assigned a mobile phone number to the support group and all the messages sent to this designated phone number were delivered to each participant involved in the study via a web interface. BulkSMS©, a company in South Africa, provided the application-to-person (A2P) messaging service necessary for mobile phones to access ZygoHubs© software. Airtime usage was tracked using online accounts. All messages were recorded at the ZygoHubs© web interface to be used in analysis.

### Data collection and analysis

The ZygoHubs© web interface recorded all messages sent to the group mobile phone number from which investigators produced a transcript for analysis. Prior to analysis, each participant was assigned a unique identifier distinct from their mobile phone number. Consistent with the goal of exploring social support themes as part of the SMS group interaction, we excluded facilitators’ communications from analyses and focused only on interactions between participants. We used thematic analysis on all messages to identify patterns and themes that focused specifically on the social and psychological aspects of participating in the support group over the 12-week period.

Investigator triangulation (Kimchi, Polivka, & Stevenson, 1991) was used to reduce bias and thereby enhance the trustworthiness of the data. The analysis involved four investigators with different backgrounds and areas of expertise with respect to professional discipline, cultural background, and qualitative research experience to ensure triangulation and attention to the cultural context in which the study took place. Data was also reviewed at different time points after the support group had finished. First, the data were de-identified, such that each participant was assigned a unique identifier, and line numbers were inserted in the text. Five authors were involved with analysing the data. One of the authors read the transcript in full before starting the open coding process and making memos in the margins, which became the basis of coding and data analysis. This and another author met several times until they reached a consensus on the coding system. One of these authors then proceeded with coding the SMS messages. Next, a third author independently reviewed the transcript and the coding system to identify portions of text that fell within each identified code. The authors’ results were compared to identify areas of agreement and disagreement. Any disagreement in the completeness of the coding system or in coding of the SMS messages was resolved through discussion between the three authors. Because most members of the research team who analysed data were not South African, a fourth author from South Africa, who is familiar with the cultural background of the participants, read the transcripts for cultural context and language, and reviewed the coding of the SMS messages. Her suggestions for additional coding themes were discussed to reach agreement. Finally, a different author reviewed the coding system and transcript to ensure that adjacency pairs (e.g. utterances that are paired in a question-response or statement-response manner) were captured within each code. These adjacency pairs served to demonstrate the co-construction of social support. Upon further review of the initial open coding, the authors felt the identified themes fit well within Simoni et al.’ (2006) social support model as presented above.

### Ethical considerations

The Kopano project was ethically approved by The University of Pretoria. Participants gave informed consent and could withdraw from the study if they wished to. Much effort was made to ensure anonymization of data and maintaining of confidentiality between respondents. Because of the sensitive nature of the discussions the researchers developed a protocol of steps to follow in case of situations where a participant may be at risk of harm (e.g. self-harm, suicide ideation) or if there is a risk of harm to others. The steps include discussion within the research team or external experts to determine the appropriate course of action; contact with the participant involved to clarify issues and to determine if further action is needed. If so, the participant is referred to appropriate agencies or professional services.

## Results

Participants sent 734 text messages over the course of the 12-week intervention (*M* = 61.2 messages per week). The number of text messages sent ranged from 4 to 229 per participant (Median = 84) over the course of the intervention. The overall level of participation varied between participants, and was significantly influenced by whether the individual experienced technological problems with her handset. Of the seven participants, five were active throughout the intervention sending between 65 and 229 total SMSes during their enrolment. Participant 7 was very active during the week she was enrolled, sending 44 SMSes, before withdrawing from the project. Participant 4 sent only five SMSes over the course of the intervention but confirmed on multiple occasions that she was reading all the messages and felt involved in the group. The researchers, as the facilitator, sent 207 SMSes and the mentor sent 76 SMSes over the course of the intervention (see Table 1 for enrolment and participation by number of SMS messages and social support domains discussed).

**Table 1.** Enrolment and participation by number of SMS messages and social support domains discussed.

Participant	Weeks of enrolment	Number of full weeks enrolled	Enrolled, technology problem-free weeks	Total messages sent	Average messages sent per enrolled, technology problem-free week	Domains of support discussed
1	1–12	11	11	188	17	AES; IS; SS; AD; GS
2	1–12	11	9	84	9	AES; IS; AD
3	2–12	10	9	229	25	AES; IS; SS; AD; GS
4	3–12	9	9	5	0.5	GS
5	3–12	9	9	120	13	AES; IS; SS; AD; GS
6	3–12	9	9	65	7	AES; IS; AD
7	3–5	1	1	44	44	AES; AD
Mentor	2–12	10	10	76	8	AES; IS; AD
Clinician	1–12	11	11	207	19	AES; IS; AD

Notes: AES = Appraisal and Emotional Support; IS = Informational Support; SS = Spiritual Support; AD = Acceptance and Disclosure of HIV status; GS = Gratefulness for the support group.

Most messages were sent between 6:00 am and 4:00 pm Monday through Friday. No messages were sent between midnight to 4:00 am. The researcher, as the facilitator, monitored the group around-the-clock for the 12-week intervention. There was significant fluctuation in group activity from day-to-day without a clear pattern. There were three discussions in which all the members were involved over the course of a day or several days. Other times, there were extended lulls in the conversation during which neither the mentor nor the clinician could engage the group with questions, topic suggestions or greetings. Frequently, two-way dialogue between a single participant and a clinician would take place. All participants had the experience of this type of exchange at least once over the course of the group. However, almost all messages directed at the group or an individual by another participant would receive a response by at least one other group member. The clinician’s difficulty redirecting discussion or assuring that all members were involved was obvious in three instances: first, initially one member would send multiple messages back-to-back which consisted of unrelated questions; secondly, when a participant sent a message that was irrelevant to a current discussion; thirdly, when participants requested information which had been previously discussed in detail. It should be noted that we did not see a pattern that the number of messages a participant sends related to their levels of satisfaction with the SMS support group.

From these messages, which were the content of the support group discussions, we identified five social/psychological themes: appraisal and emotional support, informational support, spiritual support, acceptance and disclosure of HIV status, and gratefulness for the support group (see Table 2). Within each theme examples are provided below to demonstrate how participants co-constructed the system of support by acting interchangeably as both providers and recipients.

**Table 2.** Social/Psychological themes.

Category	Theme	Common topics
Appraisal and Emotional Support	Serostatus disclosure	Seeking and offering support
	Relationship difficulties	How to disclose and to whom
	Negative affect	Care
		Concern
		Encouragement
		Advice
		Negotiating partners’ HIV testing
		Expression and management of emotional distress
Informational Support	Medical concerns	Advice
	HIV and Pregnancy Myths	Information exchange
		Pregnancy health behaviours
		HIV transmission during pregnancy and delivery
		Taking ARV
		HIV and breastfeeding
		Diet and physical health
Spiritual Support	Coping	Spirituality and coping
		Encouraging faith and prayer
		Religiosity as comfort
HIV Acceptance and Disclosure	Coping with HIV status Stigma	Managing grief
		Denial
		Mistrust of health care system
		Openness and acceptance
		Stigma associated with HIV diagnosis and barrier to disclosure
Gratefulness for the Group	Benefits	Group assisted with coping
		Built courage to disclose
		Gained valuable information
		Helped to manage suicidal ideation

### Appraisal and emotional support

Participants used the group to share thoughts and to elicit support about highly stigmatised, personal, and sensitive information. Elicitations for support ranged from direct questions (e.g. ‘what must I do?’ [Participant 12]) to merely expressing one’s inner thoughts (‘my husband’s love reduced when we found out that we are positive’ [Participant 9]). Important topics around which women sought and provided appraisal and emotional support consisted of serostatus disclosure, relationship difficulties, and general negative affect.

#### Serostatus disclosure

Many conversations focused on the issue of disclosing one’s serostatus to others, with women seeking input from the group on how to proceed after discovering they are HIV-positive. The interchange between Participants 4 and 6 provides an example of one woman seeking help from the group regarding who to share her serostatus with and how this call for help elicited supportive and practical responses from the group:

What must I tell my patner [partner] about my status. or my family. [Participant 4]

you must tell the truth about your status because your family & partner will support you in every step you take. [Participant 6]

Such advice was not helpful to other women, as Participant 7 highlights negative expectations of disclosing her serostatus: ‘U [You] know wat [what] now I am scared my boyfriend gonna [going to] live [leave] cuz [because] he negative’.

#### Relationship difficulties

Women sought social support when feeling isolated from others, particularly from their romantic partners. They turned to the group to express frustrations, to share their emotions, and to seek advice about managing problems in their relationships. Additionally, a number of participants sought help from the group because they felt unable to convince their romantic partners to get tested for HIV. In response, group members created an environment that showed care for one another while also providing encouragement. This process is illustrated by the following exchange:

My husband didn’t go 4 [for] a test as promised, what must I do. [Participant 12]

Give him time his not ready. [Participant 9]

Try 2 [to] encourage yr [your] husband 2 [to] go to e [the] tests. [Participant 7]

Talk to him, go to canceler [counsellor] and ask atvice [advice]. [Participant 4]

Portions of the conversation also demonstrated the extent to which HIV status and relationship dynamics can impact mental health, and the care that participants had for one another around these issues:

How must I cope knwng [knowing] dat [that] my hubby is neg [negative] n [and] im pos [positive]. But we were both neg [negative]. Did I brough [brought] dis [this] illness into de [the] house? How? Never mind in just an hour I won’t be living in dis [this] earth. I cnt [can’t] live lyk [like] this. Gud [Good] bye every1 [everyone] til [until] we meet again. [Participant 12]

Don’t stress so much it usually happen u [you] are not the cause on most people if happen if he understand. Please don’t feel like everyone is rejecting be strong and pray tell hiv that I am going to beat u [you] and no one will know that I am with you. [Participant 11]

#### Negative affect

The support group provided a venue for participants to express negative affect, both related to the struggles of living with HIV and general life stresses. One woman expressed serious negative feelings related to suicidal ideation [Participant 12]. Other women used the group as one might use a close friend, sending messages that convey a general sense of malaise without any specific request for help:

I am feeling dawn [down] today am feling [feeling] depresed [depressed]. [Participant 9]

I am not ok I am about 2 [to] give up everything it’s not well. [Participant 7]

In response, participants created an environment that showed care for one another while also providing help and encouragement. Specifically, group members probed for more information so that they could tailor their support to meet their peers’ needs [e.g. ‘wat’s [what’s] going on’ (Participant 7) ‘why do u [you] feel like that, wha [what] happend, [happened] is something u [you] cant share’ (Participant 3)]. This concern that group members showed for one another persisted over time, with participants checking in days after one woman shared her internal struggles by asking ‘how do u feel 2day [today]?’ (Participant 3). Women also expressed collective concern for participants who had sought emotional support from the group and provided general affirmations and encouragement to one another:

We are warried [worried] abaut [about] you. [Participant 9]

Hang in there. We are thinking about you. [Participant 12]

Love your self. trust yourself and love the way you are. [Participant 4]

You can live many more with hiv as long as you take care of yourself. [Participant 4]

Let’s reliz [realize] all our stresses a start a new life gud [good] day. [Participant 7]

### Informational support

Nearly all women participated as either recipients or providers in conversations regarding an exchange of information. Specific topics included medical concerns and myths related to HIV and pregnancy.

#### Medical concerns

The women in the group elicited information and expressed distress relating to health behaviours while pregnant, HIV transmission during pregnancy and birth, taking ART and breastfeeding with HIV. Questions related to diet and physical health while pregnant were common, demonstrated by Participant 4’s line of texts: ‘I want to no [know] that what food I must eat. I don’t want to loose [lose] weight what must I do. I have the problem of heartburn what must i drink or eat’. In addition to the medical advice provided by the group facilitators, group members offered helpful responses to their peers by drawing on their own experience [e.g. ‘4 [for] heartburn u [you] mst [must] drink mo [more] milk o [or] Gaviscon’ (Participant 6)].

SMS conversations demonstrated that women were concerned about mother-to-child HIV transmission during pregnancy and childbirth, but also apprehensive about taking ART to prevent transmission. Many participants expressed concerns about transmitting HIV to their children, as is illustrated by Participant 7 asking ‘Ok is it possible day [that] my baby won’t be affected I am 3 months pregnant a [and] my boy friend is negative. guyz [guys] can u [you] pliz [please] help me 2 [to] find something 2 [to] do so dat [that] I won’t be stressed’. This same participant later sought additional reassurance from the group ‘is it possible that the baby is going to be negative whilst you are sharing the same blood for 9 months?’ Apprehension about ART was evident, even given the women’s strong desire to prevent HIV transmission to their children [e.g. ‘Is it possible 2 [to] have a negative baby even if u [you] dont [don’t] take Arvs’ [antiretroviral drugs] (Participant 6)]. Participants expressed concern about taking ART [e.g. ‘My dear am just scared because i have to start medicatio [medication] and i dont know how is qoinq to react on my body am five month preqnent [pregnant] and i need to prevent the baby’ **(**Participant 9)] and some attempted to survey the group for information and seek input about group members’ experiences. An interaction between two participants demonstrates the distress associated with treatment for HIV, specifically the concerns mothers have about the impact of treatment on their unborn children:

so u guyz [guys] ua [you] taking arvs [antiretroviral drugs] or not yet. [Participant 7]

not yet [taking ARVs] am scared a u [you] taking them. [Participant 6]

no I am waiting 5 [for] my 4cd [CD 4] count I don’t wat [want] 2 [to] do now doesn’t ey [they] affect e [the] baby. [Participant 7]

Participants demonstrated an awareness that HIV transmission can occur after birth, particularly through the act of breastfeeding. Some were curious if breastfeeding was an option for them [e.g. ‘I want to no [know] about breathsfeeding [breastfeeding] after the baby s [is] born’ (Participant 4)] and others expressed their concerns about how HIV will impact caring for their newborn [e.g. ‘I am stress becoz [because] iam positive and iam preg [pregnant] i don’t know if its good to breastfeed’ (Participant 11)]. In response, group members shared their understanding of breastfeeding while HIV+ [e.g. ‘if ur [you’re] positive u [you] dnt [don’t] have 2 [to] breastfeed coz [because] da [the] milk can affect da [the] baby’ (Participant 6)]. When inaccurate, group facilitators corrected participants, ensuring that women could make informed decisions about child rearing.

#### Myths related to HIV and pregnancy

Misinformation about HIV was prevalent in participants’ messages, including their questions for the group and responses to others. These communications highlighted the need for trained medical professionals to facilitate the group and correct misinformation. Four women discussed myths they learned and brought these questions to the group to ask for others’ experience and advice. Some myths were innocuous while others reflect pervasive and harmful beliefs that have been implicated in the spread of HIV. For example, some women were curious about the truthfulness behind dietary recommendations, particularly when they had been told that ingesting specific foods or beverages might adversely affect their unborn children. The following exchange provides an example of how informational support from peers may result in reinforcing myths:

is it dangerous 4 [for] da [the] baby if ur [you’re] eating ice cubes every minute. [Participant 6]

adults ey [they] always say it’s not good to eat things like dat [that] coz [because] u [you] are going 2 [to] suffer during yr [your] deliverance. [Participant 7]

However, the benefits of discussing these myths with others, including corrections from trained medical professionals, is demonstrated by Participant 7’s later question for the group: ‘Is it true dat [that] wen [when] u [you] postv [positive] u [you] a not allowed 2 [to] drink cold drinks?’ This participant seemed invested in a medical myth earlier in her group experience, but began to question things she had been told and turned to the group with the expectation that facilitators and group members would provide accurate information in a supportive manner. Participant 9 posed a question to the group reflective of the virgin cleansing myth, which is common in South African communities and has been implicated in the spread of the disease (Leclerc-Mandlala, 2002): ‘is it corect [correct] that if you sleep with a virgin your cdf [CD4 T-cell count] goes high?’

### Spiritual support

Spiritual and religious importance was apparent in the text communications of three participants. These women sent a total of 11 messages with spiritual tones, showing that they turn to their spirituality in times of distress and encourage others to have faith. Examples of turning to prayer for their own struggles include the following:

do our baby come out neg [negative] after taking this medication i pray to god eveyday [everyday] for that miracle. (Participant 9)

I am open because God is the one who no [know] my life. (Participant 4)

At other times, these women offered religiosity as a comfort to others. Participant 4 frequently encouraged women to turn to spirituality and prayer to cope with the uncertainties associated with being pregnant and HIV+:

guys lets read PSALM 41 together all chapter God will help us

Pray everyday God will give us a power because he love us,

let’s pray together at twelf [twelve] oclock pm and am. GOD WILL ANSCER [answer].

In response to two women who expressed desires to end their lives, Participant 9 offered spiritual guidance: ‘Kiling [killing] yourself is not a solution trust god he will save you he will gide [guide] you in to the right direction god loves you’.

### Acceptance and disclosure of HIV status

Engaging in and adhering to HIV treatment requires a level of acceptance that one has been diagnosed with HIV as well as the potential negative outcomes that HIV may cause. Women illustrated varying degrees of acceptance regarding their HIV status, evidenced by text messages from six participants and others’ responses to their ambivalence. Participant 11 summarised how learning of one’s positive serostatus can lead to a grief response: ‘For the first time I did have fear and guilty I was scared and angry on the same I was like going mad’. Other women were not convinced of their positive status and expressed frustration with their medical care due to perceptions of stigma. This exchange between Participants 4 and 6 demonstrates the difficulty of accepting one’s status and how others, who have likely reached acceptance, can be supportive:

as for now I am not believe that I am ptv [positive] because I am good person. [Participant 4]

gu md ppl cud b pstv 2 bt if u dnt blyv make sure u know where u stand [good people could be positive too but if you don’t believe make sure you know where you stand]. [Participant 6]

Denial of one’s status can be so pervasive that some women disagree with the test results that were reported to them:

Im not convince enough dat [that] im positive. De [The] last tym [time], me n [and] my hubby tested negative [negative]. So now i want de [the] hospital to give me the results from lab. I dont trust that thngs [things] of them dat [that] they use. Do u trust them? [Participant 12]

This participant went on at length about the HIV testing process, stating that she felt nurses performing these tests were waiting for people to be infected and reacted with shock when patients did not have HIV. She was also angry at how quickly positive test results were accepted in comparison to negative test results:

What im saying is if u [you] tested positive, they cnt [can’t] wait to tel [tell] u [you]. But once u [you] tested negetive [negative] they become suprise [surprise] dat [that] how … The first tym [time] i tested, i was negative [negative]. Guess what did they say. Cum [Come] back after 3mnths [3 months]. After ol [all] i was negetivi [negative] again. Why they did'nt [didn’t] do the same thng [thing] when they found out dat [that] im positive. To make sure that i am +. [Participant 12]

On the contrary, participants who had accepted their diagnosis tended to promote openness and acceptance for others in the group:

Just look yr [your] way forward 4get [forget] about e [your] past 90% of e [the] world is now pstv [positive]. (Participant 7)

You must tell the truth about your status because your family & partner will suport [support] you in every step you take. (Participant 6)

Some communications reflected perceptions of stigma associated with their diagnosis, which served as a barrier to disclosure: ‘Im depreset [depressed] because I have a tween [a preteen or a young teenager] and I find it difficult to share my status with him’ (Participant 9). Group members provided supportive and practical responses to these women, for example: ‘Telling him about yr [your] status is not a must unless yr [your’re] read [ready] and he has clear infomation [information] about HIV’ (Participant 3). Similar to surveying the group about ART, women asked others about disclosing their status, as demonstrated in the following communication:

I you open about your status. I you tell your parents and family. [Participant 4]

no I’m scared. [Participant 7]

when I are you not ready is fine is your choise [choice]. [Participant 4]

### Gratefulness for the support group

Five women sent messages to the group reflecting gratefulness for having been a part of this group. These participants noted that having the support of the group helped them cope with fear of their HIV positive status, build courage to disclose their status to friends or relatives, and taught them valuable information about managing HIV and having a healthy pregnancy. In general, these statements reflected a sense of family between participants, providing them with emotional support and information when they did not have others in their life with whom to have these important interactions. For example, *‘*Thanks guys it is so good to be in a good family like this ones love you all’ and ‘it is so good to have you guys in my life thanks for your surport [support]’ (Participant 9). In the most extreme case, one woman expressed gratefulness for the group in helping to address suicidal intent: ‘I can see dat [that] dis [this] group helped me so much coz [because] even my sista [sister] she was not trusting me coz [because] i was about 2 [to] hang myself, now i am comfortable we are sharing ideas’ (Participant 7).

## Discussion

This project was designed to explore if a multi-way SMS-based support group using mobile phone technology, can decrease social isolation and provide HIV-infected pregnant women with social support to address personal, social and interpersonal barriers to PMTCT treatment adherence.

All women in the support group contributed to the SMS conversations and frequently responded to peers’ questions and statements. Qualitative analysis of the social and psychological content of the SMS messages resulted in five psychological/mental health and social support themes and suggested that women benefitted from the group. The themes were: appraisal and emotional support, informational support, spiritual support, acceptance and disclosure of HIV status, and gratefulness for the support group.

Although appraisal support (affirming statements, encouragement, feedback, and modelling from supportive others) and emotional support (caring, listening, and empathic companionship) have been conceptualised as distinct constructs (e.g. Simoni et al., 2006), they appeared intertwined in participants’ messages. Specifically, their conversations showed that women cared for one another and thus wanted to offer help. Women provided encouragement and empathy to one another around the issues of disclosing one’s HIV status, problems in romantic and familial relationships, and managing sadness and hopelessness. Appraisal support can be instrumental to promoting PMTCT adherence by means of increasing individuals’ self-efficacy, defined as the ‘belief in one’s capabilities to organize and to execute the courses of action required to manage prospective situations’ (Bandura, 1995, p. 2). Self-motivation and self-efficacy are strong facilitators of PMTCT utilisation across the care continuum, from antenatal clinic attendance to infant highly active antiretroviral therapy initiation (if HIV positive) (Onono et al., 2015). In conjunction, emotional support helped to address participants’ negative affect – particularly depression and suicidal ideation – through empathy, affirmations, encouragement, and expressions of care. Previous research showed that depression and suicidal ideation is prevalent among pregnant women living with HIV and is associated with poor maternal and infant outcomes (Brittain et al., 2017; Rodriguez et al., 2017). Two of the seven group participants expressed suicidal ideation, and in response, group members provided an environment of care and concern. This indicates that group facilitators should have some training to address mental health problems, and there needs to be a risk management and referral processes to help women in need of further assessment and intervention.

Informational support (advice, provision of facts, and guidance about HIV disease, ART regimens, and adherence strategies) enhanced knowledge of HIV and ART through provision of facts. Further, it was surprising to see participants contributing knowledge above that provided by the group facilitators. Women turned to the group to seek information when feeling distressed about medical decisions and exchanged information about medical concerns, such as health behaviours while pregnant (i.e. diet and physical health), HIV transmission during pregnancy and birth, taking ART during pregnancy and the potential impact on their babies, and breastfeeding with HIV. Information exchange was not always helpful, and in some cases the group provided a means to perpetuate commonly held myths. For example, participants encouraged one another to avoid cold drinks and ice, although there are no medical recommendations consistent with this. The potential spread of misinformation supports the need for a trained professional to provide accurate information. Importantly, women saw this group as a forum to raise doubts about information they have been provided elsewhere and seek accurate responses.

Spiritual support (praying with an individual, encouraging spiritual coping, or suggesting there is a sacred purpose or larger meaning in life) emerged both as individual women’s coping techniques and as recommendation and encouragement to other group members. Women engaged in spiritual support by asking others to join in prayer, acknowledging a greater purpose, and taking comfort in the concepts of faith and miracles. Prior research has found that spiritual support can strengthen one’s self-efficacy in managing an HIV diagnosis (Simoni et al., 2006) thereby indirectly improving ART adherence. The present results indicate that SMS support groups are multi-faceted and include spiritual components that may facilitate PMTCT.

An additional type of support related to the issue of acceptance and disclosure of HIV status. Participants in the support group were recently diagnosed with HIV and many were therefore in a process of accepting their diagnosis and grieving the loss of their ‘healthy’ self. Some women were in clear denial and distrusted the medical tests that led to their diagnosis. Others grappled with reconciling their diagnosis with their belief that good people do not get HIV. Alternatively, some participants modelled acceptance of their HIV status. This modelling likely served to help reduce internalised stigma (e.g. being HIV positive means one is inherently bad), promote status disclosure, and promote medication adherence, consistent with prior support group research (Tumwikirize & Mokoboto-Zwane, 2016). The extant literature and the text messages from this study suggests that disclosing one’s HIV status is difficult because of the fear of negative consequences. Thus, the women turned to the group to express fears that disclosing would have negative consequences, or generally to ask to whom to disclose and what to say to others who do not yet know of their HIV diagnosis. A supportive SMS group potentially can address the complex relationship between social networks (friends, spouses) and HIV-positive individuals, which due to difficult dynamics within the relationship (ability to build trust, understanding and supportive disclosure, and debunking stigma) does not always lead to adherence (Atukunda et al., 2017).

Finally, most participants expressed gratefulness for having been a part of the group, indicating that the SMS intervention was successful in maintaining connection to other pregnant women living with HIV and was a beneficial intervention because of the several types of social support received. In addition, women’s statements of gratitude provided direct evidence that participation helped to reduce negative affect, promote disclosure, and improve self-efficacy.

The use of mobile phones in HIV care with adults in South Africa is widespread and largely focuses on reminders of appointments, alerts to take medication, educational messaging, monitoring of guideline compliance and data collection and direct voice communication (Aranda-Jan, Mohutsiwa-Dibe, & Loukanova, 2014; Catalani et al., 2013). However, there is a dearth of communication services that provide mobile phone support networks aimed at increasing social support and ultimately adherence. Compared to the study of Dean et al. (2012), which focused primarily on the medical knowledge participants gained from their participation in Project Kopano SMS support group, our analysis was designed to explore the psycho-social aspects of the SMS support group, specifically the ways in which social support was provided and received by group members. The findings from this study are similar to those found in a similar study, Project Zumbido, done in Mexico (Mapham, 2008). The 40 participants in Project Zumbido reported having better social networks, feeling less isolated, and having better relationships with their family members after the three-month intervention (Mapham, 2008).

Based on the Information – Motivation – Behavioural (IMB) model – the theoretical model guiding this intervention – information and motivation (i.e. social support regarding adherence, social isolation, internalised stigma, and peer and socio-cultural norms for adherence) and behavioural skills (e.g. self-efficacy) contribute to adherence behaviours (adherence to prescribed treatments), and ultimately, to positive health outcomes (CD-4 count, health related-quality of life) (Fisher, Fisher, Amico, & Harman, 2006). Our research shows how the SMS support group contributed to information sharing and experience of various types of social support, which could eventually contribute to positive health outcomes. It was not the focus of the current study to assess the degree to which the social support the women received contributed to adherence behaviours, or positive health outcomes. It will be important in future research to assess the mechanisms through which social support affects adherence and well-being.

While we employed a rigorous qualitative data analysis process to triangulate data and to ensure the trustworthiness of the evidence we collected, the credibility of our study could have been improved. Credibility emphasises how the various participants feel about the interpretations the researcher makes (Creswell, 2014). We could have enhanced credibility by using prolonged engagement, careful observation, triangulation, peer debriefing, negative case analysis, and member checks (Barusch, Gringeri, & George, 2011).

Another limitation of the study is the small sample size. Seven women eventually participated, and one was withdrawn from the study due to thoughts of self-harm and a referral to psychiatric care. The small sample size may limit the generalizability of the findings to this particular group of South African pregnant women living with HIV. On a practical note, recruitment was challenged by the time frame in which the project was implemented. Given that one of the original purposes of the project was to assess the feasibility of SMS interactive support groups, which was previously reported on (Dean et al., 2012), and the qualitative analysis we have done the sample size was deemed appropriate based on our previous work (Mundell et al., 2012). Subsequent projects based on this pilot have enrolled many more participants in different contexts and will allow for an assessment of the generalizability of these findings.

The group described here was limited to fluent English speakers – however, their language ability was not good and could have limited their interaction. The use of English language could limit the reach of the programme. To continuously assess issues of language in which the SMS support groups are administered and access, anecdotally, many potential participants for whom English is not their mother tongue often indicate that they are comfortable to communicate via SMS in English more than their first language because the words of their first language are too long. Additionally, they have indicated that they tend to communicate via SMS with friends and family in English.

The number of text messages sent indicates that the participants used the platform provided and received support. The wide array of topics raised and discussed by women demonstrates a need for communication about HIV-specific concerns that may not be addressed in their typical social networks. In post intervention individual interviews, participants agreed that they would not have joined a traditional support group due to stigma and scheduling, which is an example of why technology can help take the place of ‘on the ground’ groups. However, future implementations of SMS support would be wise to recognise and to address challenges and limitations to this project

## Conclusion

The results presented here suggest that this multi-way interactive SMS-based support group model can provide social support and information to pregnant HIV positive women leading to a reduction in isolation and negative psychological outcomes which could result in positive health outcomes. Future implementations of SMS support would benefit from the lessons learnt in this research. The group needs to be facilitated by trained facilitators that can provide accurate information and know how to deal with emotional difficulties of women. There need to be a risk management and referral protocol to follow in case participants or others may be at risk of harm. The development and enforcement of procedures to monitor and to maintain confidentiality, and participant safety is essential. There are challenges in using this technology, including the availability and affordability of the technology and provision of technical support. Finally, groups should strive to be language-inclusive, particularly in areas with multiple national languages.
